# The Effect of Deep Frying French Fries and Two Types of Iberian Pork on the Characteristics of Extra Virgin Olive Oil and the Fried Products

**DOI:** 10.3390/foods11213394

**Published:** 2022-10-27

**Authors:** Ana Isabel Carrapiso, Eva María Otero-Bazago, José Ángel Gil-Amado, Lourdes Martín

**Affiliations:** Tecnología de Alimentos, Escuela de Ingenierías Agrarias, Universidad de Extremadura, Ctra de Cáceres s/n, 06007 Badajoz, Spain

**Keywords:** Arbequina olive oil, deep frying, oil degradation, French fries, fried Iberian pork, outdoor meat

## Abstract

Although deep frying is widely used, little is known about the effect of frying different meats on the frying oil. The aims of this study were to investigate whether the pork type influences the characteristics of the frying oil, to compare any effects with those of French fries, and to research whether the use of thermally damaged oil differentially affects those products. French fries and pork from pigs reared outdoors on acorns and grass (outdoor) or indoors on a concentrated feed (indoor) were deep-fried in either raw or previously heated olive oil. The type of product affected most color parameters, *K*_268_ and the α-tocopherol content of the oil. The frying of outdoor pork hardly affected the α-tocopherol content, whereas the frying of indoor pork and especially French fries caused a significant decrease. This suggests that the meat type should be considered when setting the frying lifespan of olive oil. Regarding the fried products, L*, moisture (only French fries) and the malondialdehyde (MDA) content (only indoor pork) were the only parameters affected by the previous oil damage. The outdoor pork was less susceptible to oxidation than the indoor pork when the oil was severely damaged. Therefore, pig outdoor-based systems based on antioxidant-rich diets might be convenient to maintain oxidation at the lowest level after frying.

## 1. Introduction

Extra virgin olive oil is obtained solely by mechanical means without any refining, so it maintains a part of the olive fruit antioxidants [1,2]. Deep frying causes a drop in the content in antioxidants and other beneficial compounds and promotes reactions such as thermal degradation, hydrolysis and oxidation [3,4,5,6], and oil–product exchanges, such as the transfer of fat, water, bioactive, and/or oxidation compounds [4,7,8,9,10]. The stability of olive oil against the damage caused by the deep frying process depends on factors such as the olive cultivar, the frying time and temperature, and the type of fried food [7,8,10,11,12,13,14]. In addition, the use of damaged oil not only increases safety issues but can also affect the characteristics of the fried foods [10,15].

So far, most studies on the deep frying process have focused on French fries, and little attention has been paid to meat [7,15,16]. In this respect, there are some studies on frying meat (reviewed in [15]), including chicken [10,17], but to our knowledge, there are no studies researching whether frying meat from pigs reared on rich-antioxidant diets provides any advantage in terms of the oil degradation and the oxidation status of the fried meat. Iberian pig is a rustic breed from southwestern Europe traditionally fed outdoors on acorns and grass in a system claimed to be more sustainable than indoor systems. Due to economic advantages, most Iberian pigs are currently reared indoors, but it is the traditional system that provides the most valued products, more expensive and with higher stability against oxidation [18]. With respect to the deep frying process, traditional pork might have advantages compared with indoor pork regarding the resulting damage to the frying medium and the pork oxidation.

The aims of this study were to investigate whether the type of Iberian pork influences the degradation of the olive oil used as the deep-frying medium, compare this with the effect of frying French fries, and determine whether the use of thermally damaged oil differentially affects those products. The hypothesis to test was that the traditional outdoor pork may have a protective effect on the frying oil compared to the indoor pork and French fries, and the use of thermally damaged oil may be less detrimental for traditional pork than for other products.

## 2. Materials and Methods

### 2.1. Olive Oil Samples and Heating Process

Six containers of 5 L of extra virgin olive oil from the Arbequina cultivar were purchased from a local factory and kept at room temperature in the dark (raw oil). To obtain thermally damaged oil, 1150 mL of olive oil was heated at 170 °C for 10 h using a 1 L domestic deep fat fryer (Ufesa Excelis Inox Compact) and left to cool at room temperature for 10 h (heated oil). The heating treatment was replicated 12 times using two identical fryers. 

Oil samples from four raw oil containers and from four heated oil replicates were transferred into 8 mL transparent glass flasks to immediately measure the instrumental color; oil samples were also transferred into 60 mL amber glass flasks to be kept in the dark at −80 °C (for the tocopherol and phenol analyses) and at 4 °C (for other analyses).

### 2.2. The Deep Frying of French Fries and Iberian Pork Sirloin

Raw or heated oil (1050 mL) was placed in a fryer and heated at 170 °C. The basket with the products (no overlapping) was then put inside the fryer, which was kept uncapped. After frying, the basket was left to drain over the fryer for 2 min. Four replicates were performed per oil type (raw and heated) and per product (French fries and sirloin from the two types of pork).

For the French fries, whole potatoes were manually peeled, washed, and cut using an electric slicer to a 6 × 1 × 1 cm size immediately before frying. In total, 30 pieces (144.1 ± 4.1 g, mean ± standard deviation) were placed in the basket and deep fried for 5 min.

Regarding the pork, eight frozen vacuum-packed Iberian sirloin samples from two rearing systems characterized by a different fatty acid composition (see [19,20] for differences) were used: pork from pigs fattened in an indoor system based on a conventional concentrated feed (indoor) and pork from pigs fattened in an outdoor system based on acorns and grass (outdoor). The sirloin samples were unfrozen and sliced into 1 cm medallions using an electric slicer immediately before frying. They were placed in the basket (141.9 ± 3.9 g for the indoor sirloin; 140.0 ± 4.0 g for the outdoor sirloin) and deep fried for 3 min.

Immediately after the fried products were drained, their instrumental color, moisture, and texture were analyzed. The samples were frozen at −80 °C for the analyses of the fat content and thiobarbituric acid reactive substances (TBARS).

After frying, the oil was left for 10 h to reach room temperature. Oil samples from each replicate were then transferred into 8 mL transparent glass flasks to immediately measure the instrumental color and into 60 mL amber glass flasks to be kept in the dark at −80 °C (for the tocopherol and phenol analyses) and at 4 °C (for the other analyses).

### 2.3. Instrumental Color Measurement

The instrumental color of the oil and the fried products was measured using a Minolta Chromameter CR-300 (0° observer angle and D65 illuminant) and the CIE L*a*b* system. Chroma (C*) was calculated as (a*^2^ + b*^2^)^0.5^ and the hue angle (h°) as arctg (b* × a*^−1^). For the oil samples, five measurements were taken in transparent glass flasks (6 cm height) filled with 8 mL of oil and placed on a white surface, and the mean value was used in the data analyses. For the fried products, the instrumental color was measured immediately after frying and weighing. The measurement was repeated 10 times per batch, and the data were averaged for statistical analysis.

### 2.4. Oil Quality Parameters and Anisidine Value

The free acidity, peroxide value (PV), and the extinction coefficients *K*_232_ and *K*_268_ were determined according to the European Commission Regulation 2568/91 [21] and subsequent modifications. The anisidine value (AV) was determined according to the UNE-EN ISO 6885:2008 standard [22]. The analyses were performed twice per sample, and the sample mean was used in the statistical analysis. The free acidity was determined only in four samples of the raw oil to confirm the declared values, and the other analyses were performed on all of the samples.

### 2.5. The α-Tocopherol Content of Oil

The method reported by Inarejos-García, Gómez-Rico, Salvador, & Fregapane [23] was used. Oil was dissolved in hexane, and then the solution was analyzed using a 1100 series Agilent Technologies High Performance Liquid Chromatograph (HPLC) equipped with a silica gel Lichrosorb Si-60-5 column (250 mm × 4.6 mm i.d., particle size 5 μm; Sugerlabor) and a fluorescence detector (Agilent Technologies 1200 series, Waldbronn, Germany) with excitation and emission wavelengths set at 290 and 330 nm, respectively. The flow rate of the solvent (hexane:2-propanol, 98.5:1.5) was set at 1 mL min^−1^. Quantification was performed using the calibration solutions of α-tocopherol (Sigma, Steinheim, Germany).

### 2.6. The Phenolic Compound Content in the Oil

The procedure was based on the method described by Gómez-Rico, Salvador, La Greca, & Fregapane [24]: 2.5 g of oil dissolved in 6 mL of hexane was applied to a SPE column (diol-bonded phase 500 mg/6 mL; Supelco, Bellefonte, PA, USA) previously conditioned with 6 mL of methanol and 6 mL of hexane. The column was then washed twice with 3 mL of hexane and with 4 mL of hexane:ethylacetate (85:15). The phenolic compounds were eluted with 15 mL of methanol. The solvent was then removed under vacuum at 35 °C, and the residue was re-dissolved in 250 µL of methanol:water (1:1). The, 20 μL was injected into a 1100 series Agilent Technologies HPLC equipped with a C18 reverse phase column (250 mm × 4.6 mm i.d. × 5 μm particle size; Phenomenex, Alcobendas, Spain) at 25 °C with a flow rate of 1 mL min^−1^ and a DAD detector (Agilent Technologies G1315B, Waldbronn, Germany) set at 240, 280 and 335 nm. The mobile phase was water:acetic acid (95:5) (solvent A) and acetonitrile (solvent B), which was eluted from 97.5% (A):2.5% (B) to 77% (A):33% (B) in 50 min, following by 50% (A):50% (B) for 15 min to clean up the column. The phenolic compounds were identified by comparing their retention times with those of standards (Sigma, Steinheim, Germany, and Extrasynthese, Genay, France) analyzed under the same chromatographic conditions. Five phenols (hydroxytyrosol, tyrosol, luteoline, apigenine, oleuropein) were quantified by using the calibration solutions of the standards.

### 2.7. Frying Losses and the Chemical Analyses of the Fried Products

The frying losses were calculated by weighing the samples immediately before and after frying. Moisture was determined by desiccation at 102 °C for 24 h. The fat content was determined by solvent extraction [25]. The thiobarbituric acid reactive substances (TBARS) test was performed [26], and results are expressed as the malondialdehyde (MDA) concentration.

### 2.8. The Instrumental Texture of the Fried Products

The Warner–Bratzler shear force (WBSF) test was performed on the potato pieces immediately after frying, weighing, and color measurement. For the sirloin medallions, an additional cutting step with the electric slicer was included to obtain 1 cm cuboidal strips. The computer-assisted TA.XT Plus Texture Analyzer (Exponent Stable Micro Systems version 2.0.7.0) equipped with a HDP/90 platform, a 25-kN load cell (Exponent Stable Micro Systems), and a Warner–Bratzler blade (HDP/BS) at 2 mm s^−1^ to cut the samples perpendicularly was used. The WBSF test was repeated 15 times per batch, and data were averaged for the statistical analyses.

### 2.9. Statistical Analyses

A one-way analysis of variance (ANOVA) was performed to check the effect of heating on the oil parameters. A two-way (type of fried product and previous heating) ANOVA with interaction was performed on the data from the oil and from the fried products. When the effect was significant for variables with more than two levels, a post-hoc Tukey test was performed to compare the means. For the parameters with significant interaction, an ANOVA simple effects test (with the Sidak adjustment when there were more than two levels in a variable) was performed. The analyses were performed using the SPSS package (version 27, Chicago, IL, USA). The data were expressed as the mean ± standard deviation.

## 3. Results and Discussion

### 3.1. The Characterization of the Raw and Heated Oils

The values for the quality parameters of the raw Arbequina extra virgin olive oil (free acidity: 0.12 ± 0.01% oleic acid; peroxide value: 6.4 ± 0.3 mEq O_2_ kg^−1^; *K*_232_: 1.7 ± 0.0; *K*_268_: 0.11 ± 0.01) were lower than the maximum allowance established by the European Regulation 2568/91 [21] and the subsequent modifications (0.8% oleic acid, 20 mEq O_2_ kg^−1^, 2.5 and 0.22, respectively).

Table 1 shows the results obtained for both the raw oil and the oil heated at 170 °C for 10 h. The heating treatment had a significant effect on most variables (all except b*, C*, and the tyrosol content), resulting in marked changes in color, a substantial increase in the oxidative status, and a dramatic decrease in the antioxidant (namely, *α*-tocopherol and phenols) content.

Most of the instrumental color parameters (L*, a*, and *h*°) were significantly affected by heating at 170 °C, with a general increase in the values (Table 1). Previous studies on commercial blends of olive oil [27] and Arbequina and Lechin oil at moderate temperature (100 °C) also reported an increase in L*, although for the Picual oil, a decrease was found [28]. This inconsistent effect may be related to the differences among cultivars in the antioxidant content, as the oil pigments are more susceptible to thermal degradation when the antioxidant content is lower. In this respect, the increase in L* has been related to a decrease in the pigment content during heating [28], whereas for other oils, the opposite effect has been related to the formation of decomposition compounds [29]. The marked increase in a* (Table 1) was much larger than the slight trend reported at 100 °C in oil from Arbequina and other olive cultivars [28] and has been related to changes in pheophytin [26], which degrades during storage and thermal treatments [30].

All of the oxidation parameters were markedly affected by the heating treatment, with a substantial increase (*p* < 0.001 for all of them, Table 1). The anisidine value (AV), which mainly measures *α*,*β*-unsaturated aldehydes [22] formed from hydroperoxide decomposition, was the parameter with the largest variation, with a 25-fold increase (Table 1). The increase in AV was much more marked than the one reported at a lower temperature (80 °C for 24 h) for Arbequina oil [12]. Regarding PV (which measures primary oxidation products), the results match those from most studies, generally reporting a rise in PV caused by heating [12,31], although a drop attributed to the decomposition of hydroperoxydes was also reported [1,32]. With respect to *K*_232_ and *K*_268_, the results are in line with those of previous studies [1,32].

In the case of the tocopherol and phenol contents, heating at 170 °C caused a significant decrease in all of the compounds except tyrosol (Table 1). The drop was especially marked for α-tocopherol, hydroxytyrosol and oleuropein, which decreased in concentration more than seven times. The results are in line with previous studies performed on olive oil heated at similar temperatures [1,10,11,13,33].

In conclusion, the results show that heating at 170 °C for 10 h was sufficient to obtain severely thermally damaged Arbequina oil.

### 3.2. The Effect of Deep Frying French Fries and Pork Sirloin on the Raw and Previously Heated Olive Oils

The results for the raw and previously heated oils after deep frying the French fries and the two types of Iberian pork sirloin are shown in Table 2. According to the results from the two-way ANOVA with interaction, the type of fried product affected five out of the ten oil parameters included in Table 2, the previous heating affected all of them except L*, and the interaction affected four parameters (a*, *h*°, *K*_268_, and the α-tocopherol content) (Table 2). The oil variables affected by the interaction were analyzed by performing a simple effects test. It revealed that a* was significantly affected by the type of product fried, both for the oil without and with previous heating (*p* < 0.001 and *p* = 0.018, respectively). The Sidak test showed that, when the oil had not been previously heated, the oil after frying the three products (French fries, and indoor and outdoor pork) was significantly different in all cases, whereas when using previously heated oil, only the oils from frying French fries and outdoor sirloin were different from each other (Table 2). Regarding *h*°, the simple effects test revealed that it was only significantly affected by the type of product fried when using the oil without previous heating (*p* = 0.003; differences between the oils used for frying French fries and both outdoor and indoor sirloin according to the Sidak test, Table 2), but not when using the thermally damaged oil (*p* = 0.931). With respect to *K*_268_, the simple effects test revealed that it was not significantly affected by the type of product fried when using the oil without previous heating, but it was in the case of the previously heated oil (*p* = 0.362 and *p* < 0.001, respectively), with differences between the oils used for frying French fries and outdoor sirloin and also between the indoor and outdoor sirloin, according to the Sidak test (Table 2). With respect to the α-tocopherol content, the simple effects test revealed that the oil without previous heating was significantly affected by the type of product fried (*p* < 0.001; differences between all pairs in the Sidak test, Table 2), but the previously heated oil was not (*p* = 0.547).

To sum up, the two-way ANOVA and the simple effects test revealed that there were six oil variables affected by the type of product fried (a*, b*, *h*°, C*, *K*_268_, and the α-tocopherol content), of which *h*° and the α-tocopherol content were affected only when the oil had not been previously damaged and *K*_268_ only when it had been previously damaged. 

The oil in which the French fries were fried generally reached the highest values for b*, C*, and *K*_268_, whereas the oil for frying the outdoor samples reached the lowest ones. Regarding a*, it followed the trend observed for the previously heated oil, but the opposite trend was observed when the oil had no previous heating. The *h*° value and α-tocopherol content of the raw oil reached the lowest values in the oil in which the French fries were fried and the highest in the oil in which the outdoor sirloin was fried. The significant effect of the type of product fried is in line with previous results for soybean oil after frying chicken and dough [34]. 

The noticeable effect of the type of product fried on the olive oil parameters might be related to the differences in the frying time itself but also to a different behavior in the chemical reactions due to mass transfer during frying. Regarding the frying time, it was adjusted to the optimal for each type of product, namely, 5 min for the French fries and 3 min for the sirloin medallions. Since a longer cooking time causes more thermal damage, the frying time is likely to be involved in the largest values for *K*_268_ and the lowest for the α-tocopherol content in the oil used to fry the French fries. However, this does not explain the differences between the oils used to fry the indoor and outdoor sirloins, which had the same frying time. This suggests that there might be additional factors involved in the differences that affect the chemical reactions. 

In this respect, a previous study on olive oil suggested that thermal degradation and lipid migration from the food to the frying oil were the cause of the differences in some olive oil parameters after frying dough, pork adipose tissue, and salmon fillets, all of them with the same frying time [8]. In our experiments, the French fries were not pre-fried and, therefore, were poor in lipids. However, pork (both the indoor and outdoor groups) contains lipids, with a different fatty acid profile [19,20] and antioxidant content [35] depending on the rearing system, which may have influenced the susceptibility to the oil damage induced by the deep frying. In this regard, it has been reported that the outdoor pork products (and their lipids) are less susceptible to damage than the indoor ones due to the presence of natural antioxidants built up during the pig fattening period [18]. This might explain the higher α-tocopherol content in the oil that was not previously heated when the outdoor pork was fried compared with the indoor pork (Table 2). No evidence of any protective effect of this antioxidant was found when the oil was already damaged, probably because the α-tocopherol content was extremely low (Table 2). Even so, the lower value for *K*_268_ found in the previously heated oil (thermally damaged oil) after frying the outdoor pork than the indoor pork and the French fries suggests that there may be a protective effect from the outdoor pork.

Regarding the previous thermal damage of the oil itself, its effect after the deep frying process was still marked and even increased slightly in terms of the instrumental color (L*, a*, and *h*° were affected before frying, whereas all parameters except L* were affected after frying) (Table 1 and Table 2). 

The results show that the type of fried product has a marked influence on the oil parameters, which might influence its frying lifespan; the largest variations occurred when the raw oil was used to fry the French fries. The differences between the oil used to fry indoor and outdoor sirloin also revealed that the type of meat might be a relevant factor when setting the frying lifespan of olive oil as a deep frying medium.

### 3.3. The Effect of Oil Damage on the Characteristics of the Fried Pork and French Fries

Table 3 shows the results for the French fries and the indoor and outdoor sirloin medallions fried using either raw or thermally damaged (previously heated) oil. 

The use of thermally damaged oil had a slight effect on the fried products: L*, moisture, and the MDA content were the only parameters affected according to the two-way ANOVA results, with a significant interaction for the two last parameters (Table 3). The simple effects test performed on them (Table 3) revealed that the previous heating affected only the moisture of the French fries (*p* < 0.001) and not that of the indoor and outdoor sirloins (*p* = 0.897 and 0.180, respectively); the MDA content of the indoor sirloin had a *p* value < 0.001, compared with *p* = 0.865 and *p* = 0.083 for the French fries and outdoor sirloin, respectively.

Regarding the instrumental color of the fried products, L* was the only parameter affected by the previous damage of the oil. The effect was especially noticeable for the French fries (the raw oil provided less luminosity to the French fries than the previously heated oil) according to the Tukey test (Table 3). This result is in line with that from a previous study on loin chops, which showed a significant effect of the type of oil used as the frying medium on L*, as well as on the colored materials measured at 420 nm, which were attributed to differences in the development of color and the oxidative status [36]. The absence of an interaction indicates that the damage caused to the oil did not have a differential effect depending on the type of product fried.

With respect to mass transfer and composition, the effect of oil damage was slight: the frying losses and fat content were not affected (Table 2), and the moisture content was only affected in the French fries according to the simple effects test. 

Regarding the frying losses, the lack of effect of frying in damaged oil is in line with that reported for fish products [37,38]. With respect to the fat content, the lack of effect is in line with some studies on French fries [37,39]. However, it should be noted that the effect of frying French fries in damaged oil on the fat content has been generally inconsistent: an increase [40], a decrease (which authors relate to sampling issues [34]), and significant fluctuations [41] have also been reported. The inconsistency might be due to differences among the frying procedure, the oil characteristics, and the French fries themselves (cultivar, pre-frying treatments, etc.).

According to the simple effects test, the moisture content of only the French fries was affected, with the raw oil providing a higher content than the previously heated oil (Table 3). In this respect, a previous study on potatoes and hake showed a lack of effect of oil damage on the moisture content [37], although a significant effect of the oil type on potato moisture has also been reported [42]. 

The MDA content (which measures by-products from lipid oxidation) was affected by the previous oil damage only for the indoor pork according to the simple effects test, being higher in the indoor pork fried in the previously heated oil than in the raw oil (Table 3). The effect is in line with the significant effect of the oil type on the MDA content reported for fried pork loin [36]. The increase in the MDA content could be related to the absorption of thermo-oxidized oil into the product [43]. However, the lack of effect on the outdoor samples, where oil absorption should have been similar, suggests that there might be additional factors involved. In this regard, the increase in the MDA content in the indoor samples but not in the outdoor ones might be related to a larger susceptibility to oxidation in the former, as discussed in Section 3.2. The fact that the MDA content in the outdoor samples remained low even after frying in damaged oil confirms the benefit of using raw meat with low susceptibility to oxidation.

With respect to the instrumental texture, the lack of effect of the oil damage (Table 3) is in line with results from sensory studies on French fries and fish nuggets [38] and also with the lack of effect on crust thickness in French fries [39]. However, significant fluctuations (increases and decreases) in the hardness of French fries related to previous thermal damage have also been reported, although there were no significant correlations between texture and PV or AV [41], which may indicate that there is not a strong relationship between oil damage and texture.

To sum up, the thermal damage of the frying oil had a slight effect on the instrumental color and moisture (only significant for the French fries) and on the MDA content (only significant for the indoor samples). The slight effect on the instrumental color and the lack of effect on the instrumental texture suggest that most consumers might perceive no clear differences in the French fries and no differences in the pork samples despite the toxicological risk of using severely damaged oil. In addition, the results revealed that the oxidation of the fried meat varied according to the type of rearing system, suggesting that pork from systems providing antioxidant-rich diets is less susceptible to oxidation when using damaged oil.

## 4. Conclusions

The noticeable effect of the type of fried product on the oil parameters confirms that the damage caused by deep frying is not only related to the thermooxidation favored by high temperatures but also to oil–product interactions. In this regard, frying meat from pigs reared outdoors with an antioxidant-rich diet (outdoor pork) hardly affected the α-tocopherol content of the oil, whereas the frying of indoor pork (from conventionally reared pigs) and especially French fries caused a significant decrease. This suggests that the characteristics of the meat should be considered when setting the frying lifespan of olive oil.

Regarding the effect of deep frying using severely damaged oil on the fried products, the results for the instrumental color and texture showed only subtle changes, suggesting that most consumers might be unaware of the damage of the frying oil used and its health risk. In addition, the results suggest that outdoor pork is less susceptible to oxidation than French fries regardless of the oil damage and less susceptible to oxidation than indoor pork when the frying oil is severely damaged. This highlights the importance of the type of fried product and its oxidative stability due to the repercussion on the oxidative stability of the fried product, suggesting that outdoor rearing systems based on antioxidant-rich diets are more convenient to maintain oxidation at the lowest level after frying. 

## Figures and Tables

**Table 1:** Results from the instrumental color measurement, the oxidation parameters, and the α-tocopherol and phenol contents *.

	Raw Oil	Heated Oil	*P*
L*	19.4 ± 1.6	26.5 ± 3.4	0.010
a*	−1.6 ± 0.3	3.7 ± 1.7	<0.001
b*	13.8 ± 1.7	16.5 ± 2.6	0.143
*h*°	−1.4 ± 0.0	1.3 ± 0.1	<0.001
C*	13.9 ± 1.7	16.9 ± 2.7	0.114
Anisidine value (AV)	3.2 ± 0.1	82.5 ± 7.8	<0.001
Peroxide value (PV) (mEq O_2_ kg^−1^)	6.4 ± 0.3	10.2 ± 0.5	<0.001
*K*_232_	1.7 ± 0.0	10.9 ± 0.6	<0.001
*K*_268_	0.11 ± 0.01	1.12 ± 0.09	<0.001
*α*-tocopherol (mg kg^−1^)	143.7 ± 12.3	4.6 ± 0.6	<0.001
Hydroxytyrosol (mg kg^−1^)	6.1 ± 0.7	0.7 ± 0.1	<0.001
Tyrosol (mg kg^−1^)	5.9 ± 0.7	5.3 ± 0.6	0.369
Luteoline (mg kg^−1^)	3.3 ± 0.4	1.2 ± 0.0	<0.001
Apigenine (mg kg^−1^)	6.4 ± 0.4	3.8 ± 0.2	<0.001
Oleuropein (mg kg^−1^)	106.6 ± 3.7	13.5 ± 0.5	<0.001

* The data are expressed as the mean ± standard deviation. *P*: statistical significance from ANOVA applied to data for Arbequina oil before and after heating at 170 °C for 10 h.

**Table 2:** Results from the instrumental color measurement, the oxidation parameters, and the α-tocopherol of Arbequina olive oil after the frying process *.

	Raw Oil			Heated Oil			*P*		
	French Fries	Indoor Sirloin	Outdoor Sirloin	French Fries	Indoor Sirloin	Outdoor Sirloin	Product Type	Previous Heating	Int
L*	24.1 ± 0.8	23.1 ± 1.1	22.9 ± 2.5	23.8 ± 2.7	21.2 ± 1.3	20.2 ± 3.5	0.134	0.127	0.593
a*	^3^ −0.7 ± 0.2^c^	^2^ 0.2 ± 0.3 ^c^	^1^ 0.8 ± 0.3 ^c^	^1^ 6.2 ± 1.6 ^a^	^1,2^ 3.8 ± 0.6 ^b^	^2^ 3.0 ± 0.9 ^b^	0.105	<0.001	<0.001
b*	19.8 ± 0.3 ^a^	17.5 ± 2.4 ^ab^	14.2 ± 3.1 ^abc^	19.0 ± 2.9 ^a^	12.7 ± 1.5 ^bc^	9.2 ± 1.6 ^c^	<0.001	0.003	0.153
*h*°	^2^ −1.54 ± 0.0 ^b^	^2^ 0.8 ± 1.6 ^a^	^1^ 1.5 ± 0.0 ^a^	1.3 ± 0.1 ^a^	1.3 ± 0.1 ^a^	1.2 ± 0.1 ^a^	0.001	0.004	0.001
C*	19.8 ± 0.3 ^a^	17.5 ± 2.4 ^ab^	14.2 ± 3.1 ^bc^	20.1 ± 2.4 ^a^	13.3 ± 1.3 ^bc^	9.8 ± 1.3 ^c^	<0.001	0.008	0.070
AV ^1^	6.5 ± 1.4 ^b^	6.0 ± 0.9 ^b^	5.2 ± 1.4 ^b^	92.8 ± 3.1 ^a^	80.5 ± 2.2 ^a^	86.0 ± 21.6 ^a^	0.376	<0.001	0.444
PV ^2^ (mEq O_2_ kg^−1^)	9.5 ± 0.8 ^bcd^	7.6 ± 1.4 ^cd^	7.0 ± 1.0 ^d^	14.9 ± 2.5 ^ab^	12.8 ± 1.1 ^ab^	17.0 ± 5.3 ^a^	0.272	<0.001	0.141
*K*_232_	1.9 ± 0.1 ^b^	1.7 ± 0.0 ^b^	1.5 ± 0.3 ^b^	11.4 ± 1.1 ^a^	10.3 ± 1.7 ^a^	9.2 ± 3.0 ^a^	0.258	<0.001	0.497
*K*_268_	0.19 ± 0.02 ^c^	0.17 ± 0.02 ^c^	0.16 ± 0.03 ^c^	^1^ 1.23 ± 0.03 ^a^	^1^ 1.13 ± 0.08 ^a^	^2^ 0.89 ± 0.12 ^b^	<0.001	<0.001	<0.001
α-tocopherol (mg kg^−1^)	^3^ 115.1 ± 5.3 ^c^	^2^ 129.0 ± 2.1 ^b^	^1^ 140.6 ± 7.4 ^a^	4.6 ± 0.6 ^d^	4.6 ± 0.5 ^d^	4.2 ± 0.6 ^d^	<0.001	<0.001	<0.001

* Different superscript letters (abc; right) within the same row indicate significant differences at the *p* < 0.05 level in the Tukey test. Different superscript numbers (1,2,3; left) for the same type of oil indicate significant differences at the *p* < 0.05 level in the ANOVA simple effects test and the Sidak test for the parameters with significant interaction. The data are expressed as the mean ± standard deviation. *P*: statistical significance based on a two-way (type of fried product, previous oil heating) ANOVA with interaction (Int). ^1^ Anisidine value. ^2^ Peroxide value.

**Table 3:** Results for the parameters of the fried products and significance (*P*) from a two-way (type of fried product, previous oil heating) ANOVA with interaction (Int) *.

	French Fries		Indoor Sirloin		Outdoor Sirloin		*P*		
	Fried in Raw Oil	Fried in Heated Oil	Fried in Raw Oil	Fried in Heated Oil	Fried in Raw Oil	Fried in Heated Oil	Product Type	Previous Heating	Int
L*	48.2 ± 2.7 ^c^	53.0 ± 2.3 ^ab^	52.0 ± 1.3 ^abc^	55.1 ± 0.9 ^a^	50.8 ± 1.3 ^bc^	49.7 ± 1.9 ^bc^	0.004	<0.001	0.150
a*	0.3 ± 3.1 ^ab^	−1.1 ± 3.7 ^b^	4.2 ± 0.3 ^a^	3.1 ± 0.7 ^ab^	3.8 ± 0.9 ^a^	3.9 ± 1.1 ^a^	0.001	0.324	0.805
b*	28.4 ± 2.0 ^a^	28.9 ± 2.5 ^a^	18.0 ± 0.9 ^b^	15.5 ± 0.2 ^b^	14.9 ± 0.7 ^b^	16.3 ± 1.3 ^b^	<0.001	0.082	0.146
*h*°	−0.8 ± 1.5 ^b^	0.0 ± 1.7 ^ab^	1.3 ± 0.0 ^a^	1.4 ± 0.0 ^a^	1.3 ± 0.1 ^a^	1.3 ± 0.1 ^a^	0.002	0.458	0.590
C*	28.5 ± 2.0 ^a^	29.1 ± 2.3 ^a^	18.5 ± 0.8 ^b^	15.8 ± 0.3 ^b^	16.7 ± 1.5 ^b^	15.4 ± 0.7 ^b^	<0.001	0.073	0.102
Frying losses (%)	35.3 ± 3.1	34.8 ± 4.0	31.0 ± 1.9	31.8 ± 1.1	32.8 ± 1.2	34.9 ± 2.84	0.034	0.442	0.614
Fat (mg kg^−1^)	56.9 ± 8.6 ^c^	67.9 ± 10.0 ^bc^	108.7 ± 13.2 ^a^	104.3 ± 6.6 ^a^	94.8 ± 7.8 ^a^	88.2 ± 14.3 ^ab^	<0.001	0.991	0.213
Moisture (mg kg^−1^)	^1^ 721.3 ± 2.3 ^a^	^2^ 683.4 ± 8.8 ^b^	594.8 ± 25.5 ^c^	592.9 ± 8.9 ^c^	577.0 ± 4.1 ^c^	566.1 ± 13.8 ^c^	<0.001	0.005	0.033
MDA ^1^ (mg kg^−1^)	0.71 ± 0.11 ^a^	0.69 ± 0.25 ^a^	^1^ 0.19 + 0.03 ^b^	^2^ 0.61 ± 0.08 ^a^	0.21 ± 0.02 ^b^	0.29 ± 0.07 ^b^	< 0.001	0.005	0.004
Shear force (kg)	0.4 ± 0.1 ^b^	0.4 ± 0.2 ^b^	2.7 ± 0.9 ^a^	2.5 ± 1.1 ^a^	3.0 ± 0.6 ^a^	2.6 ± 0.6 ^a^	<0.000	0.624	0.562
Work of shear (kg s^−1^)	2.1 ± 1.0 ^b^	1.5 ± 1.0 ^b^	5.5 ± 1.8 ^a^	5.9 ± 1.9 ^a^	7.1 ± 1.2 ^a^	6.4 ± 1.6 ^a^	<0.001	0.810	0.497

* Different superscript letters (abc; right) within the same row indicate significant differences at the *p* < 0.05 level in the Tukey test. Different superscript numbers (1,2; left) within the same product indicate significant differences at the *p* < 0.05 level in the ANOVA simple effects test for the parameters with significant interaction. The data are expressed as the mean ± standard deviation. *P*: significance from a two-way (type of fried product, previous oil heating) ANOVA with interaction (Int) *. ^1^ Malondialdehyde.

## Data Availability

The data supporting the findings of this study are available from the corresponding author upon reasonable request.
